# Understanding the Link Between Functional Profiles and Intelligence Through Dimensionality Reduction and Graph Analysis

**DOI:** 10.1002/hbm.70149

**Published:** 2025-02-21

**Authors:** Francesco Alberti, Arianna Menardi, Daniel S. Margulies, Antonino Vallesi

**Affiliations:** ^1^ Integrative Neuroscience and Cognition Center (UMR 8002) Centre National del la Recherche Scientifique Paris France; ^2^ Wellcome Centre for Integrative Neuroimaging, FMRIB, Nuffield Department of Clinical Neurosciences University of Oxford United Kingdom; ^3^ Department of Neuroscience University of Padova Padova Italy; ^4^ Padova Neurosciene Center University of Padova Padova Italy

**Keywords:** functional connectivity, functional gradients, intelligence, interindividual differences, topology

## Abstract

There is a growing interest in neuroscience for how individual‐specific structural and functional features of the cortex relate to cognitive traits. This work builds on previous research which, by using classical high‐dimensional approaches, has proven that the interindividual variability of functional connectivity (FC) profiles reflects differences in fluid intelligence. To provide an additional perspective into this relationship, the present study uses a recent framework for investigating cortical organization: *functional gradients.* This approach places local connectivity profiles within a common low‐dimensional space whose axes are functionally interpretable dimensions. Specifically, this study uses a data‐driven approach to model the association between FC variability and interindividual differences in intelligence. For one of these loci, in the right ventral‐lateral prefrontal cortex (vlPFC), we describe an association between fluid intelligence and the relative functional distance of this area from sensory and high‐cognition systems. Furthermore, the topological properties of this region indicate that, with decreasing functional affinity with high‐cognition systems, vlPFC functional connections are more evenly distributed across all networks. Participating in multiple functional networks may reflect a better ability to coordinate sensory and high‐order cognitive systems.

## Graphical abstract

This study uses a data‐driven approach to model the association between functional connectivity variability and interindividual differences in intelligence. We describe an association between fluid intelligence and the relative functional distance of the right ventral‐lateral prefrontal cortex from sensory and high‐cognition systems.

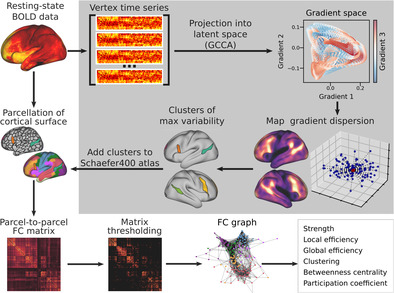

**Summary**

- Functional gradients are a new approach to model connectivity in a neurocognitive space.
- Position of frontal regions along the transmodal‐unimodal gradient is associated with intelligence scores.
- This suggests a role of frontal regions in coordinating internal schemata in response to external sensory input.

## 1 Introduction

Current neuroscience conceptualizes behavior as the result of the dynamic interaction of distributed communities of cortical regions and subcortical structures. These interactions demonstrate a characteristic functional architecture (Yeo et al. 2011) and stable topological features (Van Den Heuvel and Sporns 2013; Sporns 2011; Van Den Heuvel and Sporns 2011). However, the human brain also shows a high degree of structural and functional variability across individuals (Mansour et al. 2021), owing to the interaction of genetic and environmental factors (Burger et al. 2022).

The unique functional and structural characteristics of an individual's brain have been compared to a fingerprint, as they exhibit distinctive patterns of structural morphology (Wachinger et al. 2015), white matter tracts (Kumar et al. 2017), and intrinsic functional connectivity (FC) (Finn et al. 2015; Smith et al. 2015) that can be used to accurately identify it. These different sources of interindividual variability have been mapped across the cortex demonstrating that association areas exhibit the most unique structural‐functional patterns, making the dorsal‐attention, frontoparietal (FPN), and default‐mode (DMN) the most variable networks across individuals (Mansour et al. 2021; Mueller et al. 2013). Multiple studies have shown that it is possible to predict behavioral measures from individual resting FC organization, stressing the importance of associative regions and related networks for higher‐order cognition (Mansour et al. 2021; Finn et al. 2015; Smith et al. 2015).

The ability of resting FC to relay relevant information regarding task‐related activity (Wasmuht et al. 2018; Raichle 2015; Fox et al. 2007, 2006; Smith et al. 2009) may be partly due to it encompassing a variety of functional states during unconstrained cognition (Mckeown et al. 2020; Stoffers et al. 2015). Low frequency components of resting fMRI, specifically, have been proposed to reflect potentially separate electrophysiological and metabolic processes supporting acquisition and maintenance of contextual information (Yousefi and Keilholz 2021; Dohmatob, Dumas, and Bzdok 2020; Wasmuht et al. 2018; Raichle 2015, 2010). Slow traveling waves of activity are thought to support the spatio‐temporal organization of brain activity (Raut et al. 2021; Yousefi and Keilholz 2021) which constitutes the *functional space* within which task‐related brain dynamics unfold (Song, Shim, and Rosenberg 2023; Gao, Mishne, and Scheinost 2021). Hence, the relative location of cortical regions within this space might be associated with cognitive abilities. By adopting a low‐dimensional perspective on resting‐state activity, this work aims to provide new insight into how variability of functional organization translates into behavioral differences.

**FIGURE 1:**
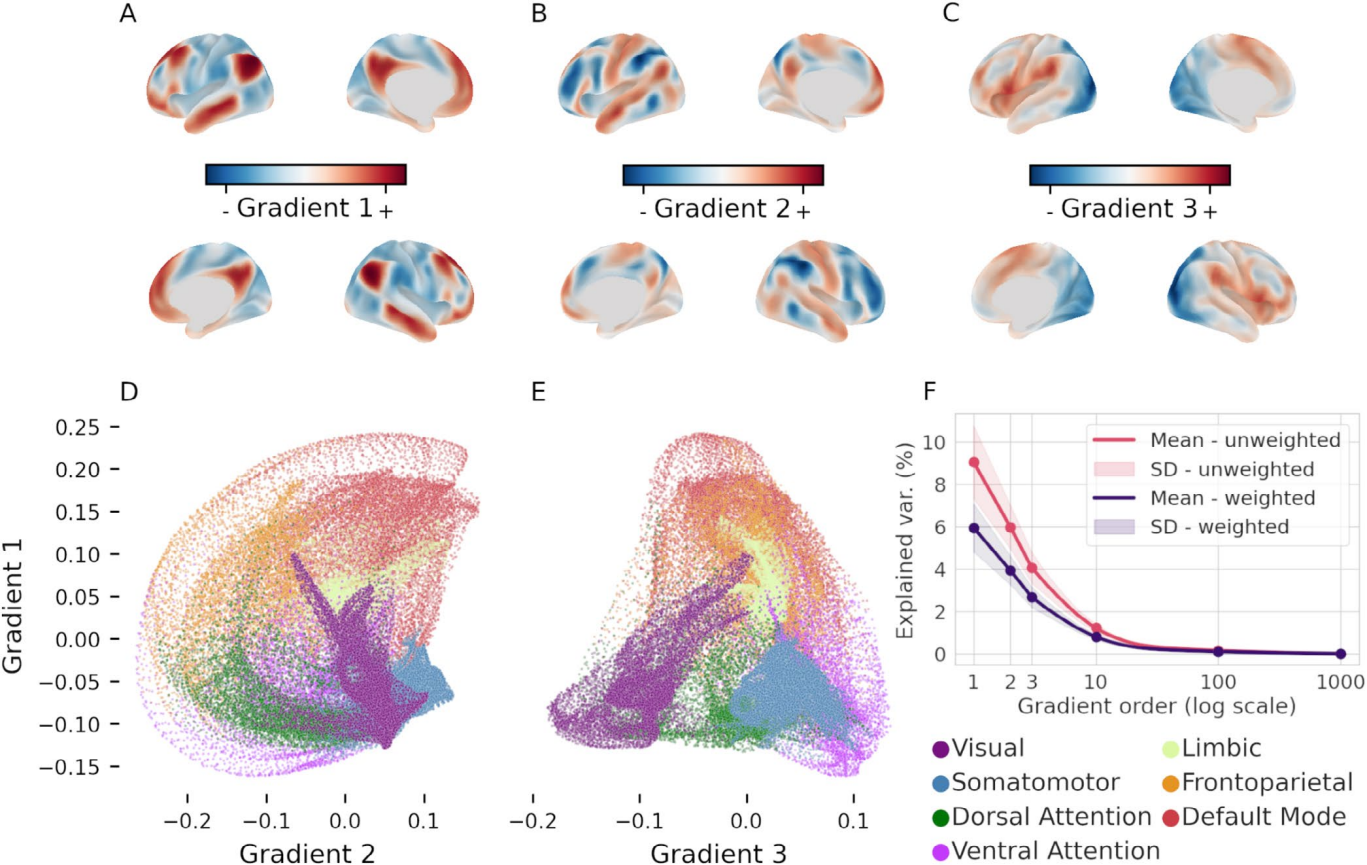
(A–C) The first three gradients of functional connectivity (group median) displayed on the inflated cortical surface. These three dimensions each recapitulate a different functional axis, as follows: Sensory‐DMN axis (A), FPN‐DMN (B), and visual‐somatomotor (C). (D–E) Scatterplot of the median location of all cortical vertices along the first and second gradient (D), and first and third gradient (E). Vertices are colored based on their affiliation to the seven resting‐state functional networks as per legend (bottom right). (F) Average scree plot of the variance explained by the first 1000 components of the individual embeddings before (pink) and after (purple) weighting by the fraction of variance explained by the group embedding of individual components.

With this objective, we adopt a framework that describes intrinsic FC patterns through a set of *functional dimensions* that reflect different axes of differentiation of brain activity (Margulies et al. 2016). This approach employs dimensionality reduction to reveal the principal components (latent dimensions) of FC data and blood oxygen level dependent (BOLD) time series (Hong et al. 2020). Such dimensions are referred to as *functional gradients* as they describe gradual transitions between maximally different functional profiles over anatomical space (Figure 1). For instance, the principal gradient captures the similarity of local FC patterns with those of the default mode network (DMN) and primary sensory areas (Margulies et al. 2016). This work examines the first three gradients as they explain the most of variance in the original data and recapitulate relatively clear functional axes (Smallwood et al. 2021). Together, functional gradients define a space that reflects the topological relationships between regions, so that regions situated close to one another have a similar BOLD time series or FC profile, reflecting the topology of the functional organization of the cortex. Locations along the principal gradient have already been associated with certain topological characteristics (Lioi et al. 2021) and FC range (Oligschläger et al. 2017). Hence, investigating the relationship between gradients and network topology could contribute to our understanding of how variability of FC is linked to cognitive performance.

Specifically, in the present study, we investigate interindividual FC variability within the functional gradient framework to better understand how it relates to different facets of intelligence. A rich debate exists around the construct of intelligence, which abilities it encompasses, and how to measure it (Duncan, Assem, and Shashidhara 2020; Deary, Penke, and Johnson 2010; Waterhouse 2006; Neisser et al. 1996; Salovey and Mayer 1990; Cattell 1943; Spearman 1904). In this context, the term intelligence collectively refers to the cognitive functions that underlie our ability to comprehend and interact with the changing environment based on contextual and prior knowledge (Neisser et al. 1996). These abilities can be grouped into two separate components—fluid and crystallized intelligence (Mungas et al. 2014; Heaton et al. 2014; Cattell 1943)—with distinguishable neuroanatomical and neurofunctional substrates (Xu et al. 2023; Santarnecchi et al. 2021, 2017; Colom, Jung, and Haier 2006). Fluid skills involve problem‐solving and adapting to novel situations, and are related to the activity of fronto‐parietal regions (Xu et al. 2023; Jung and Haier 2007). Whereas crystallized abilities are related to general knowledge acquired through experience, and rely on temporal and inferior frontal regions (Xu et al. 2023; Gainotti 2006). These and other cognitive abilities famously show high covariance and thus can be further summarized by a single measure of general intelligence: the *g*‐factor (Colom, Jung, and Haier 2006; Spearman 1904). Notably, it has been shown that individual intelligence scores are linked to the macroscale functional organization of the cortex (Menardi, Spoa, and Vallesi 2024; Gonzalez Alam et al. 2022; Mansour et al. 2021; Santarnecchi et al. 2017; Finn et al. 2015; Smith et al. 2015). This study builds on such evidence using functional gradients to synthetically characterize differences in FC in terms of relative functional affinity of regions with specific combinations of systems. This allows us to investigate if and how independent aspects of FC variability relate to facets of intelligence.

More specifically, to derive functional gradients we used generalized canonical correlation analysis (GCCA) to decompose the resting‐state BOLD time series of unrelated subjects from the Human Connectome Project (HCP), Young Adults dataset (David C. Van Essen et al. 2013). We identified the first three latent dimensions that best explain the intrinsic activity patterns across individuals. Using these three gradients, the activation profile of any given point on the cortical surface can be projected into a tridimensional space where coordinates quantify the expression of separate features of FC. Thus, to map FC variability, we estimated the dispersion in this *gradient space* for each vertex on the cortical surface sampled from every individual (Bethlehem et al. 2020). We identified eight clusters from this map where dispersion is maximal, and we separately tested the association between the three gradients at these loci and intelligence measures. This approach allowed us to test the association between intelligence and FC variability along distinct, interpretable dimensions. Specifically, we studied the effect of each of the clusters' gradients on individual scores of fluid intelligence, crystallized intelligence, and general intelligence. Then, to further understand the implications of gradient dispersion, we assessed whether gradients relevant to cognitive scores are also associated with variability of topological properties of FC profiles.

## 2 Methods

### 2.1 Participants and Data

The study was based on openly accessible data provided by the WU‐MInn HCP (Van Essen et al. 2013). Due to computational constraints, we selected a subsample of 500 individuals from the full dataset (about half the total: *N* = 1113). To avoid potentially unwanted hereditary effects on the observed variables, in case of participants coming from the same family only one was included (*N* = 338). From these we excluded those who lacked scores from one of the 10 cognitive test batteries, necessary to compute general intelligence (*N* = 2). In addition, we ensured that all participants had available four resting fMRI runs to provide more solid connectivity estimates. To monitor the potential effects of motion inside the scanner, we computed the mean framewise displacement (FD), observing an average FD across the four runs between 0.04 and 0.24 mm for all subjects. In consideration of Parkes et al. (2018) study on the effects of motion correction strategies, we concluded that our participants showed very mild degrees of movement inside the scanner, according to both lenient and stringent approaches (Parkes et al. 2018). As such, no participant was excluded based on motion. As a result, we obtained a final sample of 336 healthy, unrelated participants (*F* = 178, *M* = 158) of 28.6 years of age on average (SD = 3.6). The remaining participants who were not related to subjects in the main sample (*N* = 185, mean age (SD) = 28.6 (3.8), *F* = 93, *M* = 92) were allocated to a hold‐out sample for lockbox validation of the analyses. For all subjects, four rfMRI time series of about 15 min were available, as well as scores at several off‐scan cognitive tasks including measures of memory, attention, and executive functions. The rfMRI time series were acquired using a 3 T Siemens connectome‐Skyra using a gradient‐echo EPI sequence with 2 mm isotropic voxels, 720 ms TR, and 52° flip angle. The HCP minimal preprocessing pipeline applied to these data, as described by (Glasser et al. 2013), includes correction for spatial distortion, motion, and bias field, as well as registration to T1w image. Temporal artifacts were cleaned from the data using high‐pass filtering and independent component analysis (Glasser et al. 2016). The voxels' time series were mapped to the native surface and registered to the 32 k Conte69 mesh applying 2 mm FWHM smoothing. This process improves spatial correspondence of vertices across subjects. To account for residual anatomical variability, we applied additional smoothing of 6 mm FWHM to the functional time series when computing vertex‐wise GCCA. Smoothing was also performed prior to averaging rfMRI time series within parcels to compute FC to ensure consistency of the data analyzed across different methodological approaches.

### 2.2 Functional Connectivity Gradients

Functional gradients are latent dimensions of FC space extracted through a variety of dimensionality‐reduction algorithms (Hong et al. 2020). Cortical vertices are placed along each gradient based on the similarity of their connectivity profiles with respect to the specific feature of FC that it captures. For example, vertices within the visual and somatosensory systems will be very close on the first gradient (which develops along the sensory‐DMN axis), but very distant on the second gradient (which differentiates between visual and somatosensory networks; Figure 1D,E). To derive these latent dimensions we applied GCCA (Sorensen, Kanatsoulis, and Sidiropoulos 2021; Afshin‐Pour et al. 2012) to the four BOLD time series that had previously been normalized and concatenated. GCCA generalizes principal component analysis (PCA) across more than two data sets by finding the latent factors that maximize pairwise correlations between all sets. The specific algorithm used here initially computes the most informative principal components (Figure 1F) of each subject's BOLD time series and then applies singular value decomposition to the concatenated individual components (Perry et al. 2021). This second decomposition is used to compute projection matrices that locate the subjects' data inside highly correlated subspaces within a common space whose dimensions are the functional gradients. This approach to gradient extraction makes cross‐subject comparisons more easily interpretable, as the individual principal components are all projected in the same space. Similarly to other studies (Hu et al. 2022; Bethlehem et al. 2020), we used a three dimensional representation of FC space based on the three gradients that explain the most variance in BOLD time series (Figure 1F). Following latent dimensions account for little variability in the data and are unlikely to influence dispersion measures.

In the absence of a direct measure of explained variance of GCCA components due to its two‐step decomposition approach, we computed two separate metrics (Figure 1F). The first one is the fraction of variance explained by the latent dimensions of the individual‐level decomposition. The second one is the product of the first and the cumulative variance explained by the latent dimensions of the group‐level embedding.

### 2.3 Interindividual Variability of Functional Connectivity Profiles

**FIGURE 2:**
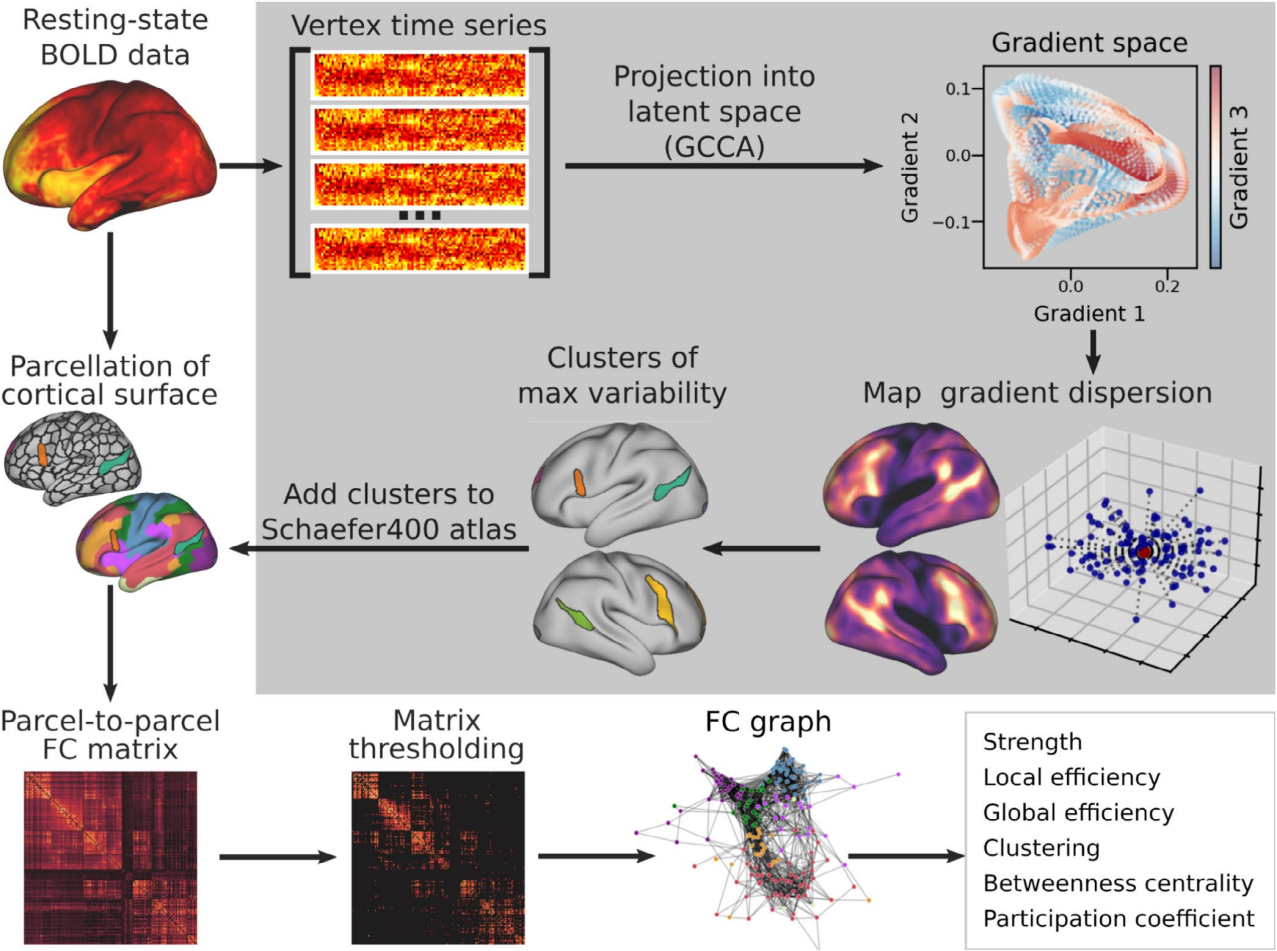
Visual summary of the analyses. The analyses are composed of two streamlines. First (gray box), we concatenated individual resting‐state time series and used GCCA to project them into a common latent space. Within this space we measured vertex‐wise dispersion maps that were then thresholded to identify clusters of maximum variability. Then (outside the gray box), we parcellated the original time series using an atlas to which the variability clusters had been added. We built a correlation matrix of regional time series and thresholded it to the top 10% of connections to obtain a graph of functional connectivity. Graph topology was then analyzed to characterize the role of different regions within it. BOLD, blood‐oxygen‐level‐dependent; FC, functional connectivity; GCCA, generalized canonical correlation.

**FIGURE 3:**
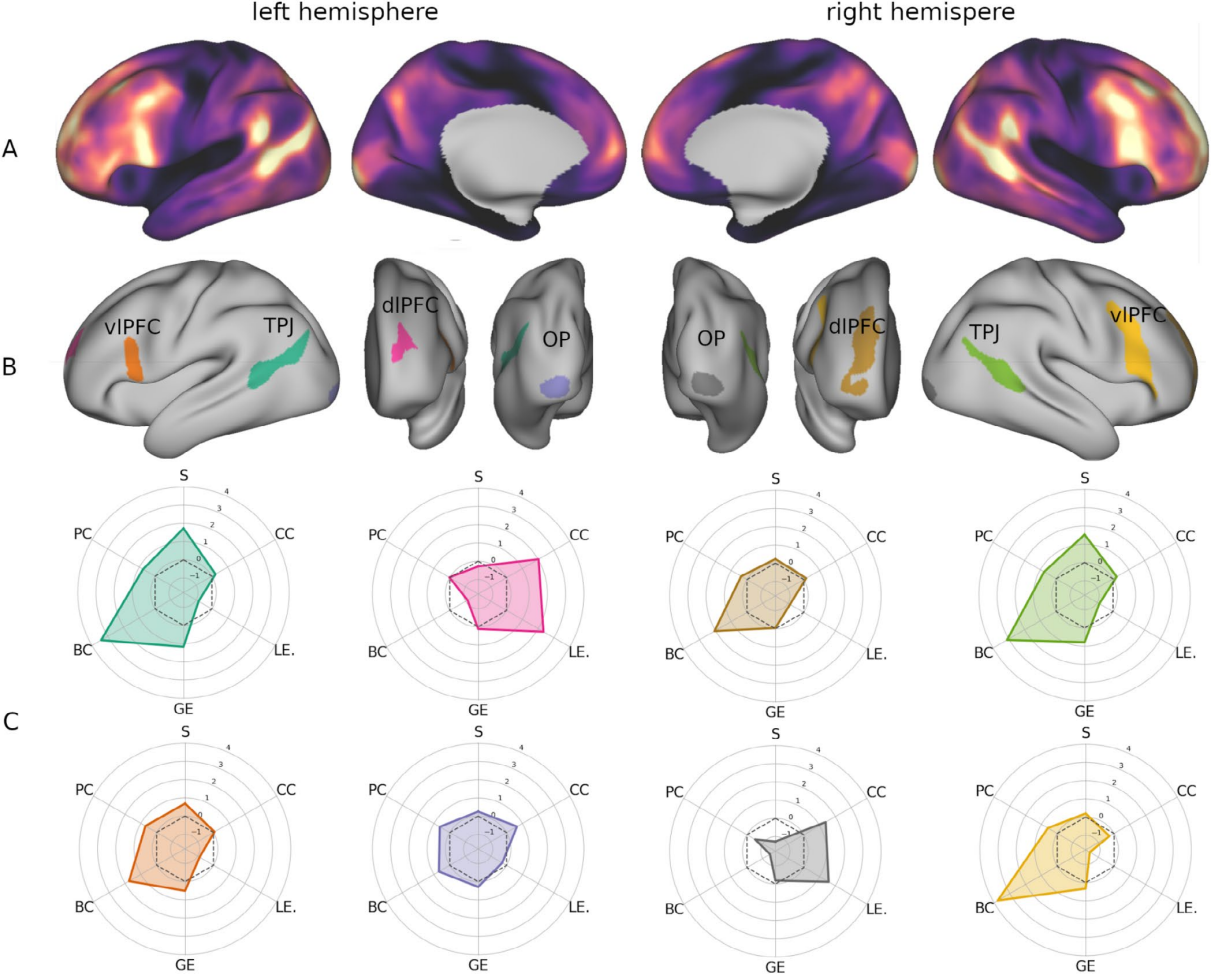
(A) Map of cross‐subject vertex dispersion in gradient space displayed on the inflated cortical surface. (B) Clusters of high interindividual variability obtained by thresholding the surface map of interquartile range at the 95th percentile. (C) Topological profiles of the variability clusters visualized as radar plots. The plots are colored based on the cluster they correspond to in panel B. BC: betweenness centrality; CC: clustering coefficient; dlPFC: dorsolateral prefrontal cortex; GE: global efficiency; LE: local efficiency; OP: occipital pole; PC: participation coefficient; S: strength; TPJ: temporo‐parietal junction; vlPFC: ventrolateral prefrontal cortex.

Interindividual variability of FC profiles was estimated by measuring the dispersion of vertices in the tridimensional space defined by functional gradients (Figure 2). Given that vertex coordinates are the three dominant latent components of FC, distance in *gradient space* reflects the similarity of their time series as captured by the gradients. Thus, a higher dispersion of vertices in gradient space can be interpreted as greater functional variability across individuals. To capture this dispersion, we adapted the procedure proposed by Bethlehem and colleagues (Bethlehem et al. 2020) to capture between‐subjects variability. To this end, a group‐level centroid was identified for each vertex in functional space. The dispersion around this point was then measured as the sum squared Euclidean distance of the individual vertices from it (Figure 3A). The same approach was also applied to determine vertex dispersion along each gradient's axis. Loci of maximum variability were identified by thresholding the functional dispersion map at the 95th percentile and applying a 200 mm^2^ minimum cluster‐size threshold, which resulted in the identification of 8 separate clusters (Figure 3B).

### 2.4 Modeling Intelligence

Three intelligence scores were computed for all subjects: crystallized intelligence, fluid intelligence, and general intelligence. Operationally, these measures were calculated by averaging multiple cognitive measures of the HCP out‐of‐scan battery. Crystallized intelligence included the picture vocabulary and the oral reading recognition tests; fluid intelligence included the dimensional change card sorting test, the flanker inhibitory control and attention test, the picture sequence memory test, the list sorting working memory test, and Penn progressive matrices; general intelligence included all the measures above as well as the pattern comparison processing speed test, variable short Penn line orientation test, and the Penn word memory test. While the first two measures were used as provided by the HCP, general intelligence was computed as the mean of its components weighted by their loadings in the factor analysis performed by Dubois and colleagues (Lohmann et al. 2021; Dubois et al. 2018) (see Table 1). To control for confounding variables, the effects of age, handedness, sex, years of education, and framewise displacement were regressed out from both intelligence and gradient measures.

**TABLE 1:** Summary statistics of crystallized intelligence, fluid intelligence, and the *g* factor, Pearson correlation between the three, and subset of task scores used to estimate them.

Cognitive component		Correlations			Test	Cognitive construct	Weight
Mean (SD)		CI	FI	GI			
General intelligence 36.49 (2.40)	Crystallized intelligence 117.5 (9.90)	—	0.38	0.79	Picture vocabulary	Language/vocabulary comprehension	0.624
					Oral reading recognition	Language/reading decoding	0.642
	Fluid intelligence 115.35 (11.61)	0.38	—	0.74	Dimensional change card sorting	Executive functions/cognitive flexibility	0.364
					Flanker inhibitory control and attention task	Executive functions/inhibition	0.259
					List sorting	Working memory	0.451
					Penn progressive matrices	Fluid intelligence	0.626
					Picture sequence Memory	Episodic memory	0.354
	—	—	—	—	Pattern comparison	Processing speed	0.232
					Variable short Penn line orientation	Spatial orientation	0.578
					Penn word memory test	Verbal episodic memory	0.294

*Note:* The task weights used for computing the *g* factor are in accordance with prior literature (Dubois et al. 2018; Lohmann et al. 2021).

Abbreviations: CI, crystallized intelligence; FI, fluid intelligence; G, *g*‐factor; SD, standard deviation.

For each intelligence measure, three multiple linear models were fit using respectively the first, second, and third gradient of the maximum‐variability clusters as explanatory variables. To address sample‐dependent biases, we implemented 10‐fold cross‐validation. The participants were split in 10 random, non‐overlapping samples stratified by the dependent variable, which were iteratively used to evaluate the *F*‐ and *t*‐values of the model trained on the remaining data. Within each fold, we computed 1000 null test statistics by randomly permuting the dependent variable. *p*‐values were calculated as the ratio of absolute null values larger than the average real statistics across folds. Correction for false discovery rate (FDR) was applied across *F*‐tests and among post hoc *t*‐tests within each globally significant model. As an additional validation test, we averaged the model coefficients across folds, obtaining a cross‐fold average model which was tested on the hold‐out sample of 185 participants. The significance of the resulting *F*‐ and *t*‐values were tested against respective distributions of 10,000 null values obtained by iteratively permuting the dependent variable and applying FDR correction as in the cross‐validation procedure.

### 2.5 Variability Clusters in Network Topology

The subjects' cortical surface was divided into 400 parcels according to the atlas by Schaefer et al. (2018), and the percentage of overlapping vertices between the clusters and resting state networks was calculated. We then added the clusters defined by our analyses to the Schaefer atlas masking the original parcels where the two overlapped, so that downstream analyses would only consider parcel vertices that were not shared with any other ROI (Figure 2). We obtained a total of 406 parcels (L: 204, R: 202), as two of the original parcels were entirely masked by the right TPJ and vlPFC clusters. To build FC graphs, a 406 × 406 adjacency matrix was computed for every individual using the Pearson's correlation coefficient between parcel‐averaged BOLD time series. All self‐connections were removed from the adjacency matrices which were then thresholded preserving only the 10% strongest positive edges in the graph to reduce the risk of including false positive connections (Figure 2). From these networks, six topological metrics of our clusters were calculated: (i) strength (total strength of all connections); (ii) global efficiency (average closeness to all the other nodes); (iii) local efficiency (average global efficiency within a node's neighborhood); (iv) clustering (the portion of a node’ neighbors also connected with each other); (v) betweenness centrality (the number of shortest paths that pass through a node); (vi) participation (how distributed a node's edges are across all communities in the graph). To calculate the participation coefficient, seven communities were defined a priori based on the seven canonical networks (Yeo et al. 2011) defined by Schaefer and colleagues (Schaefer et al. 2018). Since the variability clusters overlapped multiple networks, they were assigned to a separate community of their own.

## 3 Results

### 3.1 Functional Gradients

The first three dimensions revealed by GCCA had a spatial distribution (Figure 1) in line with the gradients previously described by Margulies et al. (2016). The principal gradient (which explains the most variance in BOLD time series; Figure 1A,F) is anchored in sensory‐motor areas and progresses toward association regions of the DMN, the second gradient (Figure 1B) differentiates between the FPN and the DMN, the third one (Figure 1C) spans from the visual to the somatomotor networks (see Figure 1D,E for a visualization of functional networks in gradient space). It should be noted, however, that the order of the second and third gradient was inverted compared to prior studies (Margulies et al. 2016; Hong et al. 2020). The first three gradients computed by GCCA showed a strong correlation, respectively, with the first (*ρ* = 0.90, *p*_FDR_ = 0.001), third (*ρ* = −0.88, *p*_FDR_ = 0.001), and second (*ρ* = −0.79, *p*_FDR_ = 0.001) FC gradient from Margulies et al. (2016).

### 3.2 Interindividual Variability of Functional Gradients

The cortical map of gradient dispersion (Figure 3A) showed a pattern in line with previous studies (Mueller et al. 2013), with peaks of interindividual variability in the dorso‐lateral temporal cortex extending to the inferior temporal lobule, and in the ventro‐lateral, dorso‐lateral, and anterior‐medial frontal cortex (Figure 3B), as well as two variability loci in the occipital poles (Figure 3B).

To understand how much variability along each gradient dimension contributed to global dispersion of FC, we tested its correlation with dispersion measured along each individual component separately.

Dispersion of the vertices' FC profiles appeared more closely related to the variability on the FPN‐DMN axis (*ρ* = 0.95, *p*_FDR_ < 0.001), followed by the sensory‐DMN axis (*ρ* = 0.85, *p*_FDR_ < 0.001), and the visual‐somatomotor axis (*ρ* = 0.60, *p*_FDR_ < 0.001). Moreover, dispersion was also positively correlated with the principal gradient (*ρ* = 0.34, *p*_FDR_ < 0.001) and negatively correlated with the other two functional axes (FPN‐DMN: *ρ* = −0.33, *p*_FDR_ < 0.001; visual‐somatomotor: *ρ* = −0.12, *p*_FDR_ < 0.001), indicating that variability was higher in the vertices near the DMN, FPN, and visual ends of the three gradients respectively.

In order to identify the loci where gradient variability across subjects is maximal, we thresholded the gradient dispersion map (Figure 3A) at the 95th percentile. From this process, eight clusters of maximum interindividual variability (Figure 3B) emerged, four in each hemisphere. Two of them were located in the bilateral temporo‐parietal junction (TPJ), two occupied the occipital poles, and the remaining four were found in the dorsolateral and ventrolateral prefrontal cortex (dlPFC, vlPFC). Two of these were located in the rostral middle frontal sulcus, and two in the opercular portion of the inferior frontal gyrus extending to the inferior frontal sulcus and middle frontal gyrus.

### 3.3 Associations Between Functional Gradients and Intelligence

After identifying the loci where interindividual FC variability was maximal, we aimed to investigate its relationship with cognitive capabilities. Thus, we examined how the location of these loci along each functional gradient related to fluid, crystallized, and general intelligence. To this end, a multiple linear model was fitted for each intelligence measure and each functional dimension using the clusters' gradients as predictors, controlling for age, handedness, sex, education, and mean framewise displacement. Since the gradients of the TPJ and occipital clusters were highly correlated between hemispheres (TPJ: first gradient, *ρ* = 0.66; second gradient, *ρ* = 0.61; third gradient, *ρ* = 0.61; occipital pole: first gradient, *ρ* = 0.94; second gradient, *ρ* = 0.96; third gradient, *ρ* = 0.97), the left and right homologs of these clusters were averaged and included as a single predictor each to avoid including redundant explanatory variables to the models.

**FIGURE 4:**
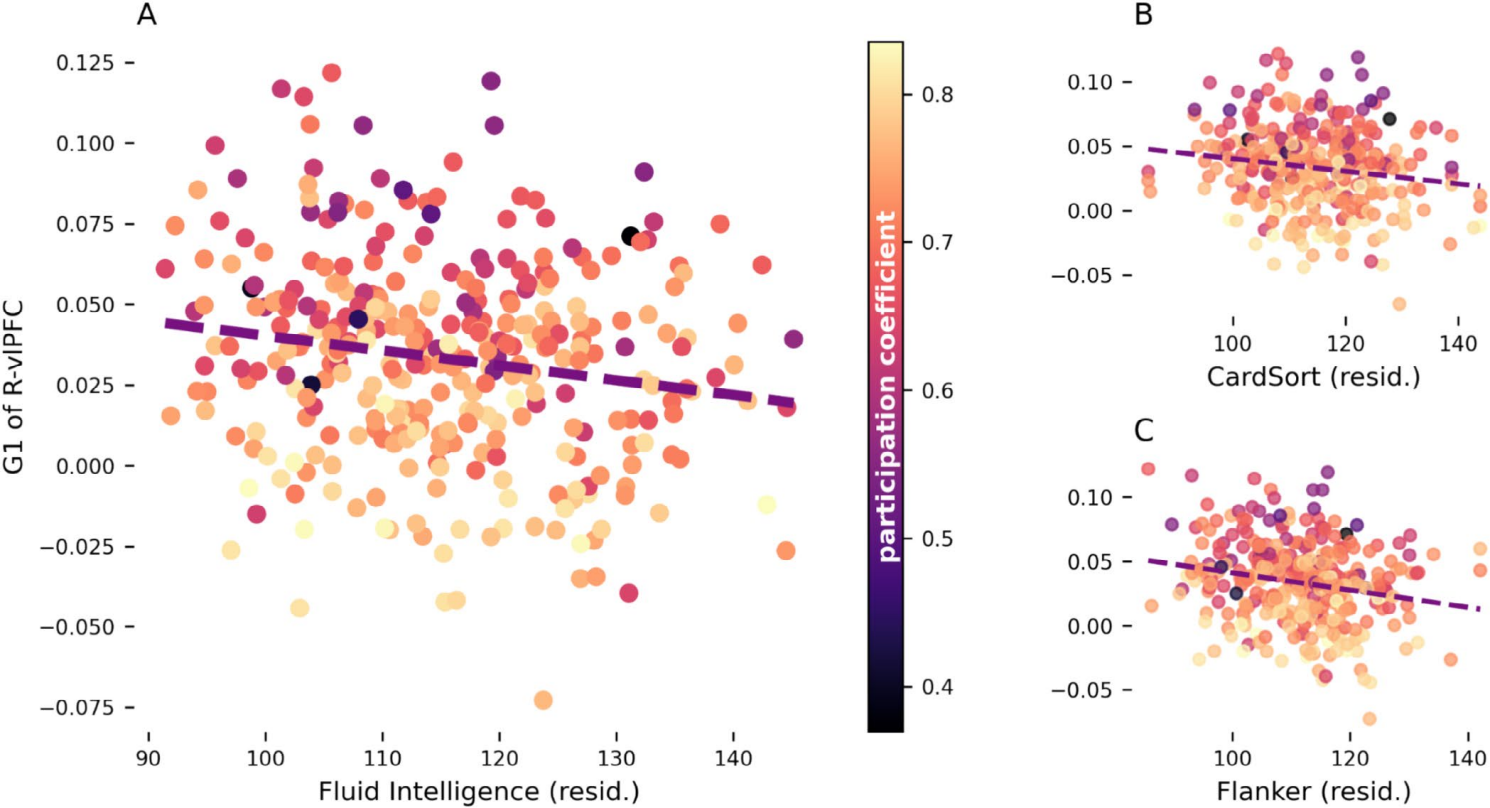
Fluid intelligence (A), card sorting (B), and flanker inhibition (C) scores plotted against the principal gradient of the right ventrolateral prefrontal cluster (R‐vlPFC) after correction for age, gender, education, and handedness. Each marker represents the principal gradient and cognitive scores of R‐vlPFC in an individual and its color represents the cluster's participation coefficient. G1: gradient 1; resid.: residuals; R‐vlPFC: right ventrolateral prefrontal cortex.

The 10‐fold cross validation procedure revealed a significant association between fluid intelligence and the principal gradient of the variability clusters (*F* = 0.38, *p*_FDR_ = 0.03). Post hoc tests revealed that this result was driven by a significant negative association between the principal gradient of the right vlPFC cluster and fluid intelligence scores (*β* = −0.16, *t* = −0.16, *p*_FDR_ = 0.03; Figure 4A). To further test the generalizability of these results, we also tested the significance of the cross‐fold average model on the hold‐out set of 185 participants (unrelated to the main dataset). The results, however, did not reach significance (*F* = 2.01, *p*_uncorrected_ = 0.06). Multiple factors could have contributed to this outcome. The considerably smaller sample size, for example, may have reduced the statistical test's power as recent studies have suggested (Marek et al. 2022). For the same reason, the GCCA components computed on the hold‐out dataset may be more biased, hindering the model's applicability to the new data.

To obtain a more cognitively detailed understanding of our results, we tested the Spearman correlation between the principal gradient of the vlPFC cluster and scores at the individual tasks included in the fluid intelligence measure (correcting *p*‐values for FDR). Moderate, significant associations were found with the dimensional change card sorting task (*ρ* = −0.15, *p*_FDR_ = 0.023; Figure 4B) and the flanker inhibitory control and attention task (*ρ* = −0.19, *p*_FDR_ = 0.002; Figure 4C). Overall, these results indicate that when the right vlPFC is functionally further from the DMN and closer to sensory systems (i.e., lower principal gradient values), individuals perform better in tasks assessing executive control.

### 3.4 Topological Properties of the Variability Clusters

The final set of analyses used graph theory to investigate the topological properties of the clusters and whether they can contribute to explain why the principal gradient of vlPFC links to fluid intelligence. To this end, a set of topological properties was computed for each cluster of high gradient dispersion. As shown in Figure 3C, this topological profiling revealed that the right vlPFC is, at the same time, the cluster with the highest betweenness centrality (*z* = 3.78) and lowest local efficiency (*z* = −1.54) and clustering (*z* = −0.29). Other clusters showed a similar pattern but not as pronounced. For example, the temporal clusters did show a high centrality (L: *z* = 3.42, R: *z* = 3.11), however their local efficiency was not as markedly low (L: *z* = −0.85, R: *z* = −0.87). The topological properties of the right vlPFC suggest that it may have a role in mediating communication between functionally segregated systems.

We then tested the association between the various topological metrics of right vlPFC and its principal gradient using FDR to correct for multiple comparisons. Significant negative correlations emerged between the principal gradient and the region's participation coefficient (*ρ* = −0.53, *p*_FDR_ < 0.001), global efficiency (*ρ* = −0.17, *p*_FDR_ = 0.002), and betweenness centrality (*ρ* = −0.12, *p*_FDR_ = 0.046). These results corroborate our previous interpretation that, when this region is functionally closer to sensory and attentional systems, it acquires a more central role in network topology with connections more broadly and evenly distributed across a larger number of networks. No other topological property of this cluster varied together with the principal gradient across individuals.

## 4 Discussion

This study suggests that the vlPFC placement between sensory processing and abstract cognition systems might contribute to explaining interindividual differences in intelligence. Regression modeling revealed that individual fluid intelligence scores are associated with the position of the right vlPFC along the functional axis going from sensory areas to the DMN. Through graph‐theory analyses we corroborated the hypothesis that vlPFC benefits from a unique connectivity profile, with a set of edges broadly distributed across multiple functional systems.

The spatial distribution of cross‐individual gradient dispersion observed in this study is analogous to that of FC variability measured through traditional connectivity analyses, with interindividual differences peaking in TPJ, vlPFC, and dlPFC (Mansour et al. 2021). In particular, the pattern of gradient variability captured by our analyses is in line with previous publications indicating associative regions as the most variable across individuals throughout multiple imaging modalities (Finn et al. 2015; Mansour et al. 2021; Kumar et al. 2017; Mueller et al. 2013). This is likely a consequence of their prolonged postnatal development (Petanjek et al. 2011; Hill et al. 2010b), which makes their connectivity and morphology less subject to genetics and more susceptible to variable environmental influence (Brun et al. 2009; Burger et al. 2022). For instance, sensory projections differ less between individuals compared to long‐range fiber tracts (Bürgel et al. 2006), which support long‐distance FC distinctive of association areas (Oligschläger et al. 2017). The differences in long‐range connectivity are thought to also underlie the high variability of cortical folding patterns that characterizes association regions (Hill et al. 2010a; Van Essen 1997). Recent studies, however, demonstrated that cortical topography and spatial relationships also have a strong influence on functional organization (Pang et al. 2023; Leech et al. 2023). In particular, a large portion of the variability of individual FC profiles is attributable to differences in spatial arrangement and topographical overlap of cortical parcels (Bijsterbosch et al. 2018, 2019). The principal functional gradient itself shows a clear spatial organization as sensory regions are located furthest from DMN (Margulies et al. 2016; Smallwood et al. 2021). It cannot be ruled out, therefore, that part of the FC variability observed here and in previous studies (Finn et al. 2015) is due to topographical differences across individuals.

Together, these sources of variability determine interindividual differences in the spatio‐temporal patterns of resting activity, which are thought to guide the functional organization of the cortex (Bolt et al. 2022; Yousefi and Keilholz 2021; Raichle 2015; Raut et al. 2021). This variability can be understood as differences in the location of cortical regions in the functional space that hosts both resting and task‐related activity (Song, Shim, and Rosenberg 2023; Gao, Mishne, and Scheinost 2021). Its main three dimensions have been consistently found to represent transitions between sensory systems and DMN, DMN and FPN, and visual and somatomotor networks (Song, Shim, and Rosenberg 2023; Bolt et al. 2022; Gao, Mishne, and Scheinost 2021; Yousefi and Keilholz 2021; Hong et al. 2020; Margulies et al. 2016). In our analyses, the principal (sensory‐DMN) gradient was the only one to significantly relate to intelligence, despite the gradient dispersion map being more closely related to variability along the DMN‐FPN axis. However, given that the principal gradient explains the most variance in intrinsic activity (Margulies et al. 2016; Hong et al. 2020), it is conceivable that interindividual variability along this dimension would better relate to individual cognitive abilities. In line with existing literature, such variability was specifically associated with fluid intelligence and executive control. This relationship was driven by a negative association between these cognitive measures and the principal gradient of the right vlPFC. That is, a larger functional segregation between this region and the DMN benefits executive functions. A possible interpretation of these data are that moving further from the DMN (in gradient space), the vlPFC acquires an intermediate topological position on the principal gradient between this network and sensory/attention systems, thus acting as a junction between them. This hypothesis was corroborated by the topological profiling of the cluster, which exhibits, on average, high betweenness and low local efficiency. That is, the vlPFC is traversed by the shortest paths between many node pairs, but it is linked to regions that are loosely connected with one another (Rubinov and Sporns 2010). Thus, this region may act as a connector hub coordinating otherwise highly segregated systems, likely the sensory and DMN ends of the principal gradient. In line with this interpretation, further analyses confirmed that the FC profile of the right vlPFC displays a higher global efficiency, betweenness centrality, and participation coefficient when it is less functionally similar to the DMN. Overall, these data indicate that this region's connections are distributed across a higher number of networks when it is located closer to the sensory end of the principal gradient. Considering the influence that cortical geometry has on FC measures (Bijsterbosch et al. 2018, 2019), this result could also indicate that a more median placement on the principal gradient is associated with being evenly distanced from all systems. Either these properties may be instrumental in guiding efficient interaction and interchange between externally‐ and internally‐driven processing: a key factor in successful executive control. Multiple studies have shown that during tasks probing executive functions, both attentional systems and DMN are alternately recruited based on which of these two processing modalities is required at any given time (Murphy et al. 2018, 2019; Konishi et al. 2015; Smallwood et al. 2013; Vatansever, Menon, and Stamatakis 2017). These studies used protocols very similar to the card sorting and flanker tasks used in the HCP dataset, which are the components of fluid intelligence most closely related to the principal gradient. Hence, the placement (be it topological or topographical) of the right vlPFC between modality‐specific and transmodal systems may interact with this specific aspect of executive functioning.

Interestingly, we did not observe any significant interaction with the measure of crystalized intelligence. One possible interpretation of this phenomena is that the neural correlates of crystallized intelligence tend to be more spatially localized, especially over the temporal lobes and prefrontal cortex, reflecting the neural correlates of storage and retrieval of information from long term memory (Lee, Choi, and Gray 2007). This is in contrast with the broader distribution of cortical regions linked to fluid intelligence, which taps into several different executive functions, resulting in highly distributed activity patterns (Santarnecchi et al. 2017). As such, we hypothesize that cognitive measures that involve more widespread systems may be more sensitive to the global functional organization captured by gradients. Furthermore, the first gradient is particularly well suited to the investigation of fluid intelligence, since prior studies have observed a critical role of DMN connectivity and interindividual differences with this measure (Santarnecchi et al. 2017; Finn et al. 2015). Finally, it is worth noting that given the characteristics of our sample (healthy young individuals between 18 and 35 years of age), a greater standard deviation was observed in measures of fluid intelligence compared to the other two measures (see Table 1). This was accentuated after regressing out the covariates (fluid intelligence, SD = 8.03; crystallized intelligence, SD = 0.10; *g*‐factor, SD = 2.02), as crystallized intelligence is thought to be more sensitive to factors such as education (Mungas et al. 2014; Heaton et al. 2014).

The main limitation of this study lies in the lack of replication of the cross‐validation results in the lockbox validation set. This discrepancy makes the degree of generalizability and reliability of the results uncertain, highlighting the need for future research to replicate our findings. For instance, the lack of replication prompts for further investigation regarding the statistical power needed to reliably observe brain‐behavior associations when relying on functional gradients. Possibly, the power of the analyses may have been hindered by the considerably smaller size of the lockbox sample (Marek et al. 2022). It would also be useful to assess the influence of sample size on GCCA components, as it could affect the bias of the group‐level decomposition. Altogether, although beyond the scope of this paper, a better understanding of how these factors may affect the methods used in our analyses is crucial to better determine the solidity of this study. The limited age range of our sample represents another limitation leaving the open question of how intelligence relates to FC gradients in younger or older populations, as well as on larger cohorts, especially in light of the methodological challenges of brain‐behavior association studies (Marek et al. 2022). Futhermore, fMRI has inherent drawbacks including its limited temporal resolution and its nature as an indirect measure of neural activity. Finally, the selection of the parcellation atlas (Schaefer 400 in this case) represents an additional researcher's degree of freedom. Although highly used in the literature, future studies might investigate the impact of using a different parcellation, especially in terms of resolution, which may affect the graph‐theory measures (Fornito, Zalesky, and Bullmore 2010).

## 5 Conclusions

In conclusion, this study expands our current knowledge on interindividual variability of the functional architecture of the cortex by decomposing it into interpretable neurocognitive axes intrinsic to individual functional patterns. Our results suggest that the link between individual functional brain fingerprint and high‐level cognitive performance might be associated with the ability of prefrontal cortices to coordinate internal schemata in response to external sensory input.

## Conflicts of Interest

The authors declare no conflicts of interest.

## Data Availability

Data used in this study can be found in the Human Connectome Project (HCP)—Young Adult repository, made available by the WU‐MInn HCP initiative (https://www.humanconnectome.org/). Codes used for the analysis are available in GitHub (https://github.com/alberti‐f).
